# Advancing towards contemporary practice: a systematic review of organisational performance measures for non-acute health charities

**DOI:** 10.1186/s12913-019-3952-1

**Published:** 2019-02-22

**Authors:** Richard Colbran, Robyn Ramsden, Karen Stagnitti, John W. Toumbourou

**Affiliations:** 1 New South Wales (NSW) Rural Doctors Network, Hamilton, NSW Australia; 2 School of Health and Social Development, Deakin University, Melbourne, VIC Australia; 3 School of Psychology, Deakin University, Melbourne, VIC Australia

**Keywords:** Organisational performance measurement, Non-acute healthcare, Non-government, Non-profit, Charity

## Abstract

**Background:**

Organisational performance measurement is a recognised business management tool and essential for survival and success. There is a paucity of methodological studies of organisational performance measurement relating to non-acute healthcare charities and this study is the first to suggest a set of evidence-informed organisational performance measures for the sector.

**Methods:**

This study was designed using a two-staged approach. A systematic review of peer-reviewed journal literature between 2003 and 2016 was conducted according to the twenty-seven (27) point checklist of the Preferred Reporting Items for Systematic Reviews and Meta-Analyses (PRISMA) complemented by a thematic analysis of eligible data using a cutting and sorting technique to generate a set of common measures of organisational performance for non-acute health charities.

**Results:**

Not one study was found relating to organisational performance of non-acute healthcare charities however four records met eligibility criteria relating to non-acute or primary healthcare services with charitable fundraising capability. Three were case studies of specific organisations that related their approach to organisational performance measurement, while the fourth compared a case study organisation to a public service. Three different organisational performance frameworks and 20 organisational performance measures were used across the four studies.

**Conclusions:**

The study concluded that (1) demonstration of organisational performance is relevant to non-acute health charities; (2) organisational performance measurement is feasible in this sector; (3) an evidence-based organisational performance measurement framework for the sector has not yet been developed nor has an existing organisational performance measurement framework been adapted for the sector, although the Balanced Scorecard is likely to be an effective option and (4) five leading measures – Quality of Service; Finance; Stakeholders (Customers and Clients); People and Culture; and Governance and Business Management; could be used to determine organisational performance in these sectors. Finally, ‘Mission and Purpose’ could be explored as a potential measure. Further research to understand why there is such limited published organisational performance evidence for the sector could be useful. Case studies of organisational measurement strategies of successful non-acute healthcare charities and research into important factors for organisational performance implementation in the sector may contribute to greater uptake and knowledge dissemination.

## Background

Internationally, the nonprofit sector continues to grow in importance and visibility [1]. In health, the evolving role of non-government health services and charities is recognised by the World Health Organisation’s Civil Society Initiative [2]. Non-acute health charities form part of this broader global industry and play a significant role in providing a range of non-hospital and maintenance-care services across many health and wellbeing disciplines often in a complementary manner with other non-charity social services such as education, disability, mental health, aged care, rehabilitation, justice and welfare. Despite the broad scope of non-acute health charities, the sector is treated somewhat homogenously in the health care system as such organisations are governed independently; have a broad range of stakeholder groups [4]; have common funding options through government contracts, donations, fee-for-service and membership; are intrinsically connected by their charitable reason-for-being [3, 4]; do not have a profit motive [5]; and benefit from unique governance legislation that allows them to function with not-for-profit status eligible for donations or tax concessions.

While non-government organisations, not-for-profits and charities share service workloads with government and for-profit providers [3] an outcome of global growth has been increased competition ([4], p. 353); an escalation of concern regarding organisational effectiveness, excellence and accountability [5]; and stakeholder agreement that charities should be well governed ([3], p. 48) and held accountable ([3], p. 50). Organisational performance measurement is “essential to the survival and success of the modern business” ([6], p. 719) and is distinct from program effectiveness in that it is not the same as summed program level measures ([7], p. 477) and provides operational and benchmarking data that can guide improvement over time, rather than point in time program level measures. Demonstrating organisational performance is likely to be advantageous in the not-for-profit and charitable health care sector as there is a link between organisational performance measurement and organisational excellence ([8], p. 182) and it can demonstrate “efficiency and effectiveness in providing services” to clients [9].

The healthcare industry itself has a long tradition of extensive and detailed performance measurement [10], however it appears the focus has predominately been on hospital, acute or clinical settings and jurisdictional systems as described by Zelman, Pink and Matthias [11] and not on the nonprofit and charity health industry. The relationship between performance measurement and organisational effectiveness in nonprofit organisations is addressed in published literature [12] however the uptake of organisational performance measurement within not-for-profit sectors has been slow ([7], p. 478). There is an empirical research literature gap for organisational performance of third sector or not-for-profit primary health care service provision ([13], p. 599 and [14]) and the sector faces a “dangerous blind spot” ([15], p.35) emanating from a dearth of robust performance data for the sector which has resulted in it being poorly understood, under-supported and potentially under-valued. There is still little consensus as to “what are the best measures of performance” ([16], p. 59) nor is there an accepted standard of measuring effectiveness. The lack of published literature does not signal that organisational performance measurement is not being implemented in non-acute health charities. It may indicate that efforts have not been methodologically designed or factors for successful implementation have been retained by organisations or not reported. The organisational performance measurement process is difficult with upwards of 70% implementation failure rate [17] which reinforces the need to identify features of successful implementations ([17, 18], p. 7) so that a central repository of learning and knowledge can be used by the sector’s leaders to inform their approach for change within their organisations.

Characteristics of non-acute health charity business models may be a challenge to organisational performance measurement. The difficulties associated with the conceptualisation of organisational performance due to the financial and legal status and goals of charities, which are based on social values, were documented back in the 1980s and 1990s ([16], p. 59). More recently it is suggested that the absence of a single end product ([7], p. 477) and the presence of multiple stakeholder groups ([7], p. 477 and [19], p. 34) create further complications. These coupled with the need to balance clinical and corporate governance responsibilities, and non-profit cultures that do not encourage accountability ([20], p.16) and restrict the introduction of contemporary professional standards ([19], p. 34) are also cited.

In terms of organisational performance measurement methodology, the World Health Organisation’s 2008 European Ministerial Conference Report [21] on the use of performance measurement for health system improvement identified conceptual frameworks as a key element of stewardship responsibilities for leaders when pursuing performance measurement for health system improvement. There are a number of organisational performance measurement frameworks or tools available for practitioners identified in literature across for-profit, public and not-for-profit industry such as Balanced Scorecard, Multi-criteria analysis [22], UTASTAR [22], Public Sector Scorecard [22], Total Quality Management [23], Peer-review analysis [24] and Social Return on Investment (SROI).

The decision regarding the choice of organisational measures is reportedly one of the major considerations for framework design as the metrics chosen should relate directly to the “organisation’s planned processes and targeted outcomes” ([25], p. 11). Any theoretical framework for performance measurement requires reference points such as measures to assess success or performance to be developed ([21], p. 6). These reference points or measures are fundamental and the lack of published literature across any of the performance tools specifically relating to the measures of organisational performance of non-acute charities suggests measures for the sector have not yet been created, adapted or tested. The difficulty in determining appropriate measures that integrate accountability with sustainability ([3], p. 51), and the time consuming need for flexibility to suit individual organisations ([26], p.79), are likely to be a challenging barriers to the introduction of an organisational performance measurement model in the sector. Authors including Gurd and Gao [17], Gomes and Liddle [27], Zimmerman [25], Bisbe and Barrubes [28], and Boateng et al. [16] suggest variations to organisational performance measures for healthcare organisations or charities.

In accepting the challenges of undertaking organisational performance measurement in the non-profit charity health sector, a clear benefit for investment and risk must also be established for both pursuing sector-specific understanding and also individual organisation implementation. The reality is that for organisational performance measurement to be successful “it is essential to take into account the specificities of the sector” ([28], p. 926) and must also be tailored to suit individual organisations. Organisational performance measurement seeks to “monitor, evaluate and communicate” the extent to which an organisation meets its objectives ([21], p. 2). So while organisational performance frameworks or tools may be transferable across for-profit, public and not-for-profit industry; or for that matter, individual sector types such as acute, non-acute, primary or community health care; success factors across industry or sector types should not be assumed as being similar as no two organisations will have the identical objectives. Hence the need for creating measures to allow comparison of tailored performance indicators developed for individual organisations within a sector such as non-acute health charities.

The survival of non-acute health charities may be threatened if they do not accept the new age of corporate and service responsibilities, contemporary business management and stakeholder expectations for accountability and excellence. The identification of evidence-informed organisational performance frameworks and measures for non-acute health charities may in-part help overcome the barriers for measurement implementation in the sector, and in turn encourage demonstration of effective business strategy and client care outcomes. Creating a competitive edge through reporting organisational performance in service, stakeholder engagement, and funding could just be what the doctor ordered for non-acute health charities to sustain their programs ([20], p. 32) and enhance their prospects of long-term success and sustainability.

This study sought to answer the question: What are the important organisational performance measures of non-acute health charities? The specific aims were to (1) gauge the extent of quality methodological studies of organisational performance measurement frameworks for non-acute healthcare charities; (2) source studies from the sector which have evaluated the feasibility, validity and effectiveness of frameworks in improving organisational performance and client care outcomes; and (3) determine which measures were most valuable to assess the performance of such organisations.

## Methods

This study was designed using a two-staged approach. Firstly, a systematic review – widely accepted as a gold standard in evidence synthesis [29], was conducted according to the twenty-seven (27) point checklist of the Preferred Reporting Items for Systematic Reviews and Meta-Analyses (PRISMA) flow statement described by Moher, Liberati, Tetzlaff and Altman [30]. This review sought to source studies that identify measures that assess the performance of non-acute health charities, and studies that evaluated the feasibility, validity and effectiveness of frameworks in improving organisational performance and client care outcomes in such organisations. The PRISMA systematic review method was chosen as it is highly regarded systematic review method as evidenced by the fact it is the preferred reporting guideline of the international EQUATOR (Enhancing the QUAlity and Transparency Of health Research) Network that seeks to improve the reliability and value of published health research literature [31] and would assure the research study’s integrity and enhance transparency.

This first stage was then complemented by a thematic analysis of eligible data using a cutting and sorting technique informed by Ryan and Bernard ([32], p. 94) to generate a set of common measures of organisational performance of non-acute health charities.

### Stage 1: PRISMA compliant systematic review

#### Eligibility criteria

The study sought scholarly peer-reviewed literature, published between 2003 and 2016, available in English and full-text online relating to organisational performance measurement of non-government organisations (NGOs) in the not-for-profit (NFP) or charitable sectors that provide non-acute healthcare.

Internationally there is a wide perspective of definitions relating to non-government, not-for-profit and charitable sectors depending on the field of topic studied ([5], p. 437; [12], p. 697). For the purposes of this study, the organisation variables were defined as –

*Non-government organisations (NGOs):* organisations that are not government or public institutions. When excluding for-profit businesses, such organisations are often referred to as the ‘third-sector’ and characterised by a vision to contribute to community issues.

*Not-for-Profit organisations (NFPs):* NGOs where all of the revenue earned is utilised to pursue the organisation’s objectives. Profits are not distributed back to owners, shareholders or members.

*Charitable organisations:* NFPs that are legally eligible for donations or specific tax concessions.

*Non-acute healthcare:* the provision of non-hospital health service where the primary clinical purpose is support for a patient with impairment or activity limitation due to a health condition. Often referred to as maintenance care, non-acute patients often require care over an indefinite period following initial assessment or treatment as opposed to complex stabilisation.

#### Search information sources

Table 1 identifies the electronic databases accessed through EBSCOHost and the five primary search fields. Each search utilised variations of Boolean operators (AND, OR, * and Parentheses) and was complemented by GoogleScholar. Additional records were sourced through peer checking and inspecting reference lists. This search activity was conducted between January 2015 and June 2015, and updated in December 2016.

**Table 1:** PRISMA Stage 1 Search Strategy and Electronic Databases

PRISMA Stage 1: Identification of records	Database search strategy
	Five (5) search fields featuring variations of the key study words were targeted using EBSCOHost as the primary search platform supported by GoogleScholar. Each search utilised variations of Boolean operators (AND, OR, * for multiple character wildcard searches, and Parentheses to specify the order of search interpreptation). 1: Organisation performance of NGOs, third-sector, non-government organisations, non-profit organisations 2: Organisation performance of health services - non-acute, primary health 3: Organisation performance of health services - child and paediatric 4: Organisation performance of health services - non-profit, NPO, third-sector, non-government 5: Health organisations, paediatric health, child health, primary health, non-acute health, non-profit organisations, non-government organisations, NGOs, non-government health, non-government primary health, third sector, third sector health, third sector child health Databases utilised in search
	A+ Education, Academic OneFile, AINHAL Complete, Applied Science & Technology Source, Art Source, Arts & Humanities Citation Index, Business Source Complete, CINAHL Complete, Communication & Mass media Complete, Criminal Justice Abstracts with Full Text, Directory of Open Access Journals, Education Source, Environment Complete, ERIC, Expanded Academic ASAP, Garden, Landscape & Horticulture Index, General OneFile, General Reference Center Gold, GrnFILE, Health Policy Reference Center, Humanities Source, Historical Abstracts with Full Text, Info Trac Health Reference Center Academic, International Biliography of Theatre & Dance with Full Text, Jewish Studies Source, JSTOR Journals, JSTOR Arts & Sciences, JSTOR Arts & Sciences IV, JSTOR Arts & Sciences VI, JSTOR Arts & Sciences IX, Legal Source, Library & Information Science Source, Library & Information Science & Technology Abstracts, MAS Ultra - School Edition, MasterFILE Premier, MEDLINE Complete, MLA International Bibliography, Philosopher’s Index, Political Science Complete, PsychINFO, Science Citation Index, ScienceDirect, Social Sciences Citation Index, SociNDEX with Full Text, Social Works Abstracts, SPORTDiscus with Full Text, Urban Studies Abstracts

#### Study selection

Table 2 outlines the Study’s PRISMA flowchart indicating search strategy and process.

**Table 2:** PRISMA Flow Chart

Stage 1: Identification	Published articles identified through database search ● Phase 1: Database search strategy (*n* = 17,823) ● Phase 2: Additional records identified through peer feedback and reference checks (*n* = 217)	Records identified through five database search strategy fields - 1: Organisation performance of NGOs, third-sector, non-government organisations, non-profit organisations 2: Organisation performance of health services - non-acute, primary health 3: Organisation performance of health services - child and paediatric 4: Organisation performance of health services - non-profit, NPO, third-sector, non-government 5: Health organisations, paediatric health, child health, primary health, non-acute health, non-profit organisations, non-government organisations, NGOs, non-government health, non-government primary health, third sector, third sector health, third sector child health
Stage 2: Screening	Records screened (*n* = 18,040)	Records excluded (n = 17,780) for not meeting inclusion criteria – ● Published between 2003 and 2015 (excluded = 3354, included = 14,686) ● Scholarly peer-reviewed literature (excluded = 7409, included = 7277) ● Available in English and full-text online (excluded = 39, included = 7238) ● Abstract confirmed record related to organisational performance measurement and healthcare (excluded = 6973, included = 265) ● Removal of duplications (excluded = 5, included = 260)
Stage 3: Eligibility	Full text articles assessed for eligibility (*n* = 260)	Records excluded (*n* = 256) for not meeting eligibility criteria – ● Study relates to an organisation providing Health and Social Services (excluded = 1, eligible = 255) ● Study relates to Organisational Performance Measurement Framework including Measures (excluded = 195, eligible = 60) ● Study relates to, or makes reference to, delivery of non-acute health services = (excluded = 46, eligible = 14) ● Provides a complete Organisational Performance Measurement Framework including Measures (excluded = 6, eligible = 8) ● Study relates to organisational performance in non-government organisation/s (excluded = 8, eligible = 4) ● Study relates to organisational performance in non-for-profit or charitable organisation/s (excluded = 0, eligible = 4)
Stage 4: Included	Studies included in qualitative synthesis (*n* = 4)	

Flow of information through the phases of the PRISMA compliant systematic review a systematic review including the number of records identified, included and excluded at each phase

The goal was to assess studies of organisations that met all three organisational characteristics – NGO, NFP and charitable eligibility, which provided non-acute healthcare services.

Inclusion criteria were used in the second (screening) and third (eligibility) stages of the four staged PRISMA-flow process. The inclusion decisions were made by the lead author and critiqued by the second and third authors. Records were sought that were scholarly peer-reviewed literature, published between 2003 and 2016 available online in English and in full-text. Two-hundred and sixty (260) articles published between 2003 and 2016 were identified as relevant. Full-text assessment of these publications (Stage 3) was then undertaken using six eligibility criteria.

The authors agreed that there were no articles sourced that related solely to organisational performance of non-acute healthcare charities, however four articles met the inclusion criteria (see Table 2). Internet searches were conducted on the case study organisations identified within the eligible records to confirm their corporation status, governance structures and service types.

Before proceeding, the potential impact of sourcing only four eligible records on the study’s validity was considered. A review of literature relating to low or nil record systematic reviews – otherwise known as ‘empty reviews’, suggested they occur for a variety of reasons; however they are not uncommon and many are listed within the Cochrane Database of Systematic Reviews [33]. Empty reviews are still important although are at risk of misguided conclusions if not well constructed ([34], p. 596). Although no eligible records were sourced relating to organisational performance of non-acute health charities, as the review was PRISMA compliant, the authors chose to proceed with analysis of the four records that met the inclusion criteria as they provided the closest information to the target sector.

### Stage 2: Data collection process and theming

The eligible studies were reviewed and the following data were extracted: 1) authors names 2) publication 3) year of publication 4) country of origin 5) key search terms 6) article key words 7) organisational performance measurement framework studied and 8) organisational performance measures utilised for the framework. A quality assessment of the eligible studies was also conducted using the four categories of The Rosalind Franklin Qualitative Research Appraisal Instrument - RF-QRA ([35], p.37) – credibility, transferability, dependability and confirmability. The risk of bias was determined to be low. Each record was defined as published peer-review literature and was assessed as being trustworthy using RF-QRA by the research team.

The performance measures for each study were collated using a cutting and sorting technique ([32], p. 94) whereby quotes or expressions that related to the study aim were identified as themes. The results were tabulated into a list of repeating or similar themes from which a table of common measures were generated.

## Results

### Study selection

Table 3 identifies the four records that met the eligibility criteria and had data extracted. An RF-QRA analysis for each record is also included.

**Table 3:** List of included records

	Eligibility Criteria					Data Extraction					Record Trustworthiness
Title Author	Confirmation the study relates to organisation/s that provide non-acute healthcare services	Study provides a complete Organisational Performance Measurement Framework including Measures	Study relates to organisational performance in non-government organisation/s	Study relates to organisational performance in non-for-profit organisation/s	Study relates to organisational performance in charitable organisation/s	Service Type	Case Study Organisation	Year Published / Journal / Origin of study	Subject search terms	Article key words	Authors of this Paper Rating: Rosalind Franklin Qualitative Research Appraisal Instrument (RF-QRA)
Implementing a balanced scorecard as a strategic management tool in a long-term care organization Schalm C.^23^	Yes (mixed acute and non-acute)	Yes (Balanced Scorecard)	Yes	Yes (Confirmed by internet search)	No (Confirmed by internet search)	Wellness services / Long-term care	The Capital Care Group, Canada	2008 Journal of Health Services Research Canada	5 Medical care, strategic planning, non-profit organizations, information resources, Decision making	3 Long term care, Quality management, Organisational Strategic Planning	RF-QRA - Level 2 Credibility: Strong Transferability: Strong Dependability: Sound Confirmability: Sound
“When Doing Good Is Just the Start to Being Good”: A Possible Tool to Improve the Organizational Effectiveness of Non-Profit Health Care Organizations Mueller J.^2^	Yes (mixed primary health and non-acute)	Yes (Looking Glass Evaluation Tool)	Yes	Yes (Confirmed by internet search)	Unspecified	Primary Health Non-Profit Organisation (NPO)	Not identified	2007 Journal of Hospital Marketing & Public Relations NZ	4 Delivery of Health Care, Efficiency, Organizational Evaluation Studies, Nonprofit/standards benchmarking	5 Health care, organizations, government, Not for Profit, competition	RF-QRA - Level 2 Credibility: Sound Transferability: Strong Dependability: Strong Confirmability: Sound
Client perceptions of the performance of public and independent not-for-profit primary healthcare Laamanen R, Ovretveit J et al.^14^	Yes (mixed primary health and non-acute)	Yes (Primary Health Care attributes scales)	Yes	Yes	Unspecified	Independent not-for-profit (INPO) Primary Care Provider	Not identified	2006 Scandinavian Journal of Public Health Finland	3 Community Health Centres – standards, Patient Satisfaction, Primary Health Care - Standards	5 Acceptance, Independent not-for-profit organisation, Performance, Primary health care, trust	RF-QRA - Level 1 Credibility: Strong Transferability: Strong Dependability: Strong Confirmability: Sound
Defining, justifying and implementing the Balanced Scorecard in the National Health Service Radnor Z, Lovell B.^5^	Yes (mixed acute and non-acute)	Yes (Balanced Scorecard)	Yes	Yes	No (Confirmed by internet search)	Primary Care Trust	City Primary Care Trust (PCT) Bradford, UK	2003 International Journal of Medical Marketing UK	3 Management, Public health – Great Britain, National Health Service, Medical Care	3 Balanced scorecard, Performance management, Primary Care Trust	RF-QRA - Level 2 Credibility: Strong Transferability: Sound Dependability: Strong Confirmability: Sound

A 2008 Canadian study by Schalm [36] details the development of a corporate Balanced Scorecard (BSC) program for The Capital Care Group – a continuing care organisation. The study details strategies for balanced scorecard implementation and offers the BSC factors and indicators used by the case study organisation along with seven lessons learnt ([36], p. 13).

A 2007 study by Mueller [3] introduced the role of NFP healthcare providers and paid particular attention to their need for financial responsibility and sustainable relationships with funders. The difficulty of non-profit performance measurement is highlighted given “the diverse environment and diffuse outcome objectives of many NPOs” ([3], p.47). The study reported on a field trial of the Looking Glass Evaluation Tool with a Primary Health Organization (PHO) in New Zealand, to determine the effectiveness of its organizational activities.

Published in 2006, Laamanen et al. [13] compared primary healthcare provided by an independent not-for-profit organization (INPO) with that provided by two Finnish public municipal organisations in terms of clients’ perceptions of performance, acceptance, and trust. The study derived from the changes connected with “new public management” ([13], p. 598) involving consumer choice and production of services based on market procedures. The study offers a scale of consumer assessment and found clients of the INPO generally rated the service more positively than clients of publicly provided services.

The BSC is the focus of Radnor and Lovell’s 2003 study [37], which offered a needs analysis for organisation performance systems and an in-depth account of the BSC and its history within the UK’s National Health System. The steps of BSC development within a local Primary Care Trust (PCT) is described, along with the indicators used and a summary of lessons learnt.

### Study characteristics

Three of the studies were case studies of specific organisations (Mueller [3], Schalm [36], and Radnor and Lovell [37]) and their approach to organisational performance measurement, while the fourth compared a case study organisation to a public service (Laamanen et al. [13]). The four records originated in different countries – Canada (Schalm [36]), New Zealand (Mueller [3]), Finland (Laamanen et al. [13]) and United Kingdom (Radnor and Lovell [37]), and were published in a variety of journals between 2003 and 2008. There was a broad range of electronic database search terms and key words, with little commonality identified between each article, suggesting the terminology in the field is underdeveloped.

Each of the case study organisations delivered non-acute care, however none identified it as their sole or primary service. None identified solely as a charitable organisation, however two of the case study organisations identified partner charitable Foundations which raise funds for the organisation through private philanthropy and public donations (Schalm [36], Radnor and Lovell [37]).

### Organisational performance frameworks and measures

Table 4 identifies the organisational performance framework and the organisational performance measures for each study. Three different organisational performance frameworks were used - the Balanced Scorecard (BSC) was used in two studies (Schalm [36], Radnor and Lovell [37]), a Primary Health Care Attributes Scale (Laamanen et al. [13]) and The Looking Glass Evaluation Tool (Mueller [3]). Twenty organisational performance measures were used across the four studies and these were themed by the authors into five categories - Quality of Healthcare Service; Finance; Stakeholders (Customers and Clients); People and Culture and Governance and Business Management.

**Table 4:** Organisational Performance Frameworks and Measures

Paper	Framework	Number of Measures	Measure				
			Quality of Service	Finance	Stakeholders - Customers & Clients	People & Culture	Governance & Business Management
“When Doing Good Is Just the Start to Being Good”: A Possible Tool to Improve the Organizational Effectiveness of Non-Profit Health Care Organizations Mueller, J. *Journal Of Hospital Marketing & Public Relations* 2007; 17 (2): 45–60.	Looking Glass Evaluation Tool	6		Financial and Asset Management Fundraising Sustainability			Governance – Strategic Management Management Practices Risk Management
Client perceptions of the performance of public and independent not-for-profit primary healthcare Laamanen, R, Ovretveit, J et al. *Scandinavian Journal of Public Health* 2006; 34 (6): 598–608.	Primary Health Care attributes scales	5	Accessibility Comprehensiveness Continuity Accountability (Quality of Care)		Trust		
Defining, justifying and implementing the Balanced Scorecard in the National Health Service Radnor, Z, Lovell, B. *International Journal of Medical Marketing* 2003; 3 (3): 174–189.	Balanced Scorecard	4		Cost Perspective	Client Perspective (Themed here because doesn’t specifically call out quality of care, instead looks to utilise traditional 4 BSC perspectives)	Learning & Growth	Internal Processes
Implementing a balanced scorecard as a strategic management tool in a long-term care organization Schalm, C. *Journal of Health Services Research & Policy 2*008; 1 (13): 8–14.	Balanced Scorecard	5			Clients (Themed here because does not specifically call out quality of care) Stakeholders Community Partnerships	People, Learning & Research	Internal Processes
	BSC 2/4 (50%)	20 (AV: 5)	4 (20%)	4 (20%)	5 (25%)	2 (10%)	5 (25%)
Number of papers that refer measures in that theme			1 (25%)	2 (50%)	3 (75%)	2 (50%)	3 (75%)

## Discussion

This study completed a systematic search and has demonstrated the limited extent of literature relating to organisational performance of non-acute healthcare charities. There were no published studies identified relating specifically to charities that provide non-acute care in their service scope. No studies were found that evaluate the feasibility, validity or effectiveness of applying an organisational performance measurement framework to improving organisational performance or patient outcomes in this sector. No studies comparatively evaluated measures to identify the most valuable measures in determining performance of such organisations.

### Value and feasibility

Considering the extent of published literature supporting organisational performance in the for-profit sector, and the increasing recognition of such business tools in the NGO and NFP sectors, the lack of demonstrable evidence to support its use and development within non-acute healthcare charities is an important finding. The four eligible studies were all published before 2009, originated from different countries and utilised highly varied key words and search terms, which may reinforce the limited extent of awareness or use of organisational performance measurement in the sector.

Richard et al. ([6], p. 719) report that the capability to accurately measure and report organisational performance is “essential to the survival and success of the modern business”. Harvey and Snyder suggest “smart non-profits are finding that goal-oriented management, combined with yardsticks to measure progress, may mean the difference between success and failure” ([20], p. 14). It is therefore reasonable to suggest that organisational performance measurement for non-acute healthcare charities would likely be of value.

Radnor and Lovell found that the majority of successful organisations within a market-based economy “use PMSs (sic. performance management systems) of varying degrees of sophistication” ([37], p. 175). As the four eligible records identify case studies that demonstrate the design of frameworks and measures, it is reasonable to suggest that similar would be feasible for development for non-acute health charities.

### Organisational performance frameworks

Three organisational performance frameworks were utilised within the study’s four records (Table 3). No evidence garnered through this systematic review suggests none of those organisational performance frameworks is more suited to this sector than another, however only the Balanced Scorecard (BSC) featured more than once. The BSC is worth investigating as an organisational performance measurement framework tool for the non-acute health charity sector as it “prevails as the dominant performance measurement system in private industry” ([38], p. 427), is used in “government, and non-profit organisations worldwide” ([22], p. 104), can be applied in healthcare ([39], p. 260) and was widely referenced and utilised in records sourced that did not meet all of the study’s inclusion criteria. The other identified frameworks - The Looking Glass Evaluation Tool [3] and Primary Care Health Attributes scale [13] were not widely used.

### Organisational performance measures

The importance of tailored sector-type and individual organisational performance measures as success reference points within conceptual frameworks and the difficulty in developing organisational performance measures for non-profits has been recognised. The study did not source any comparative studies that identify the most valuable measures for the non-acute healthcare providers in NGO, NFP and charity sectors. As such, this study contributes to the body of knowledge by being the first to propose a set of evidence-informed organisational performance measures specifically for non-acute health charities.

There were 20 organisational performance measures identified within the four assessed studies (Table 4), which were themed by the authors into five common categories. These could now be tested as measures for NGO non-acute healthcare providers in the NFP or charity sectors:

- Quality of Service
- Finance
- Stakeholders (Customers and Clients/Patients)
- People and Culture
- Governance and Business Management

Three of the four studies included measures that related to Governance and Business Management. Two of those used Kaplan and Norton’s traditional ‘Internal Processes’ perspective for BSC ([40], p. 54). Mueller [3] identified governance, strategic management, management practices and risk management as key measures. Table 4 reveals factors that can be considered to broaden the range of the Governance and Business Management measures used in this sector.

The importance of stakeholders including clients or patients, was clearly demonstrated. In terms of health care, the World Health Organisation’s European Ministerial Conference Report maps important accountability relationships across seven stakeholder groups within health systems ([21], p. 2)– patients, citizens, clinician, government, profession, provider organisation, purchaser organisation; and acknowledges their different needs. The expectations and needs of each stakeholder group vary and not just in relation to patience outcomes. Subsequently, performance measures, for each group could be considered. Three of the four studies included measures relating to the engagement and satisfaction of stakeholders. In healthcare, Bisbe et al. [28] noted that some organisations have placed client and financial perspectives at the apex of their BSC “thus assigning them equal importance” ([28], p. 923). For non-profits, Gomes and Liddle’s study [27] corroborated Kaplan and Norton’s [40] suggestion that non-profit organisations should put customers at the top of their strategic maps and three of Zimmerman’s (2009) six recommended BSC measurement perspectives for NFPs related to stakeholders ([25], p. 10). The value of stakeholders or customers to charities was demonstrated by Boateng et al. [16] in that two of five recommended organisational performance measures for charities related to client satisfaction and stakeholder involvement and it was noted that “the overall performance of charities is best measured by a set of factors that reflect the multiple and diverse stakeholders associated with charities” ([16], p. 59) and that non-financial measures including stakeholder involvement are important to the performance of charities ([16], p. 59).

Quality of Service as an organisational measure strengthens attention to an organisation’s core service. Gurd and Gao reiterated that patients are “the focus of healthcare services” ([17], p. 17) and Bisbe et al. ([28], p. 922) noted that some healthcare organisations have an additional measurement perspective on clinical outcomes. Three of the four studies included performance indicators relating to service satisfaction and one specifically noted the importance of Quality of Care to organisational performance ([13], p. 600).

In terms of financial considerations for organisational performance, the issue appears to be more focused on ‘where’, not ‘if’, finance sits in a healthcare BSC. Bisbe et al. [28] discuss the “increasing resistance within healthcare organisations, especially those in the public health sector, to placing the financial perspective at the apex of the strategy map” ([28], p. 923). Two of the four studied papers included finance measures within their frameworks.

Two of the four studies identified people, learning and growth as important measures. As Kaplan and Norton ([41], p. 257) suggest organisational learning and growth are generated from people not just systems, and subsequently People and Culture was identified as a stand-alone measure outside of Governance and Business Management.

Further investigation of records that had not met all of the eligibility criteria highlighted that some non-profit health organisations seek an organisational performance measure that assesses performance against “achieving the organisation’s final objective” ([28], p. 923). Colbran et al. [14] suggested such a measure be titled ‘Mission and Purpose’ and this may have merit as inclusion as a sixth organisational performance measure for non-acute health charities.

### Study limitations

The authors identified limitations to the study that should be considered when interpreting the results and recommendations. These limitations also reinforce the need for further study. While limited source or empty reviews are defendable, the study’s primary limitation is the small number of data sources due to the fact that no studies were found that related solely to organisational performance of non-acute healthcare charities and that only four records met the inclusion criteria. Extending the systematic review criteria beyond peer-review published literature to include grey literature (sourced outside the peer-reviewed literature), or through less restrictive eligibility criteria, may have identified reports of additional eligible records. However, if the latter had been applied, the lack of literature on performance measurement in non-acute healthcare charities sector would have been lost.

### Implications for further study

The present study identifies the need for quality methodological studies of organisational performance measurement frameworks for non-acute healthcare charities. Identifying the reasons for the lack of published evidence relating to organisational performance measurement in this sector could encourage awareness and greater knowledge sharing within the sector. The present study lists frameworks and measures and a comparative evaluation of these frameworks to understand their appropriateness and inform their selection within the sector would be of value.

In terms of effective deployment, further research to understand and validate the five BSC recommended organisational performance measures, with the additional inclusion of ‘Mission and Purpose’ is recommended, along with identification and analysis of indicators to support each measure. Research to understand the important factors for organisational performance implementation within non-acute healthcare charities is also recommended. Demonstrating impact and validating the degree of value of the full-scaled deployment of a tailored set of measures utilising an evidence-based framework such as the BSC would further inform the value of organisational performance measurement to organisations in the sector.

Finally, the opportunity to utilise organisational performance measurement to support whole-of-sector development is worth considering. While acknowledging there will always be some differences due to the different strategic orientations of health care organisations, Gurd et al. [17] encouraged health care organisations to work in groups of like organisations to produce scorecards which are both comparable but meet their own strategic needs. The aggregation of sector-specific knowledge can complement health related accreditation frameworks and enable benchmarking by providing “national or state-based pictures of performance” Sibthorpe and Gardner ([42], p. 102).

## Conclusions

The nonprofit sector continues to grow globally and so too does concern regarding the effectiveness and accountability of non-government organisations, not-for-profits and charities. Organisational performance measurement is a recognised business management tool and it has been suggested as being essential for success and survival. A relationship between performance measurement and organisational effectiveness in nonprofit organisations has been identified yet uptake in some nonprofit sectors has been slow. In healthcare, non-acute health charities form part of the broader global industry and play a role in non-hospital care however there is a literature gap in organisational performance for this sector.

This study found there is a paucity of methodological studies of organisational performance measurement relating to non-acute healthcare charities and concluded that (1) demonstration of organisational performance is relevant to non-acute health charities; (2) organisational performance measurement is feasible in this sector; (3) an evidence-based organisational performance measurement framework or tool specifically for the sector has not yet been developed, nor has an existing organisational performance measurement framework yet to be adapted for the sector, although the Balanced Scorecard is likely to be an effective option and (4) five leading measures – Quality of Service; Finance; Stakeholders (Customers and Clients); People and Culture and Governance and Business Management; could be used to determine organisational performance in these sectors.

Further study to understand and validate the five recommended organisational performance measures complemented by a sixth measure - Mission and Purpose, which was identified in the broader literature, would be valuable. Further exploration to understand why there is such limited published organisational performance evidence for the sector could be useful, as would consideration as to which existing organisational performance measurement frameworks might be most successfully adapted for the sector. Case studies of organisational measurement strategies of successful non-acute healthcare charities and research into important factors for organisational performance implementation in the sector may also contribute to greater uptake and knowledge dissemination.

## Abbreviations

BSC: Balanced Scorecard
INPO: Independent not-for-profit organization (INPO)
NFP: Not-for-profit
NGO: Non-government organisation
PCT: Primary Care Trust
PHO: Primary Health Organization
PRISMA: Preferred Reporting Items for Systematic Reviews and Meta-Analyses
RF-QRA: The Rosalind Franklin Qualitative Research Appraisal Instrument
